# Polarization of Diploid Daughter Cells Directed by Spatial Cues and GTP Hydrolysis of Cdc42 in Budding Yeast

**DOI:** 10.1371/journal.pone.0056665

**Published:** 2013-02-20

**Authors:** Wing-Cheong Lo, Mid Eum Lee, Monisha Narayan, Ching-Shan Chou, Hay-Oak Park

**Affiliations:** 1 Mathematical Biosciences Institute, The Ohio State University, Columbus, Ohio, United States of America; 2 Molecular Cellular Developmental Biology Program, The Ohio State University, Columbus, Ohio, United States of America; 3 Department of Mathematics, The Ohio State University, Columbus, Ohio, United States of America; 4 Department of Molecular Genetics, The Ohio State University, Columbus, Ohio, United States of America; University of Nebraska, United States of America

## Abstract

Cell polarization occurs along a single axis that is generally determined by a spatial cue. Cells of the budding yeast exhibit a characteristic pattern of budding, which depends on cell-type-specific cortical markers, reflecting a genetic programming for the site of cell polarization. The Cdc42 GTPase plays a key role in cell polarization in various cell types. Although previous studies in budding yeast suggested positive feedback loops whereby Cdc42 becomes polarized, these mechanisms do not include spatial cues, neglecting the normal patterns of budding. Here we combine live-cell imaging and mathematical modeling to understand how diploid daughter cells establish polarity preferentially at the pole distal to the previous division site. Live-cell imaging shows that daughter cells of diploids exhibit dynamic polarization of Cdc42-GTP, which localizes to the bud tip until the M phase, to the division site at cytokinesis, and then to the distal pole in the next G1 phase. The strong bias toward distal budding of daughter cells requires the distal-pole tag Bud8 and Rga1, a GTPase activating protein for Cdc42, which inhibits budding at the cytokinesis site. Unexpectedly, we also find that over 50% of daughter cells lacking Rga1 exhibit persistent Cdc42-GTP polarization at the bud tip and the distal pole, revealing an additional role of Rga1 in spatiotemporal regulation of Cdc42 and thus in the pattern of polarized growth. Mathematical modeling indeed reveals robust Cdc42-GTP clustering at the distal pole in diploid daughter cells despite random perturbation of the landmark cues. Moreover, modeling predicts different dynamics of Cdc42-GTP polarization when the landmark level and the initial level of Cdc42-GTP at the division site are perturbed by noise added in the model.

## Introduction

**Figure 1:**
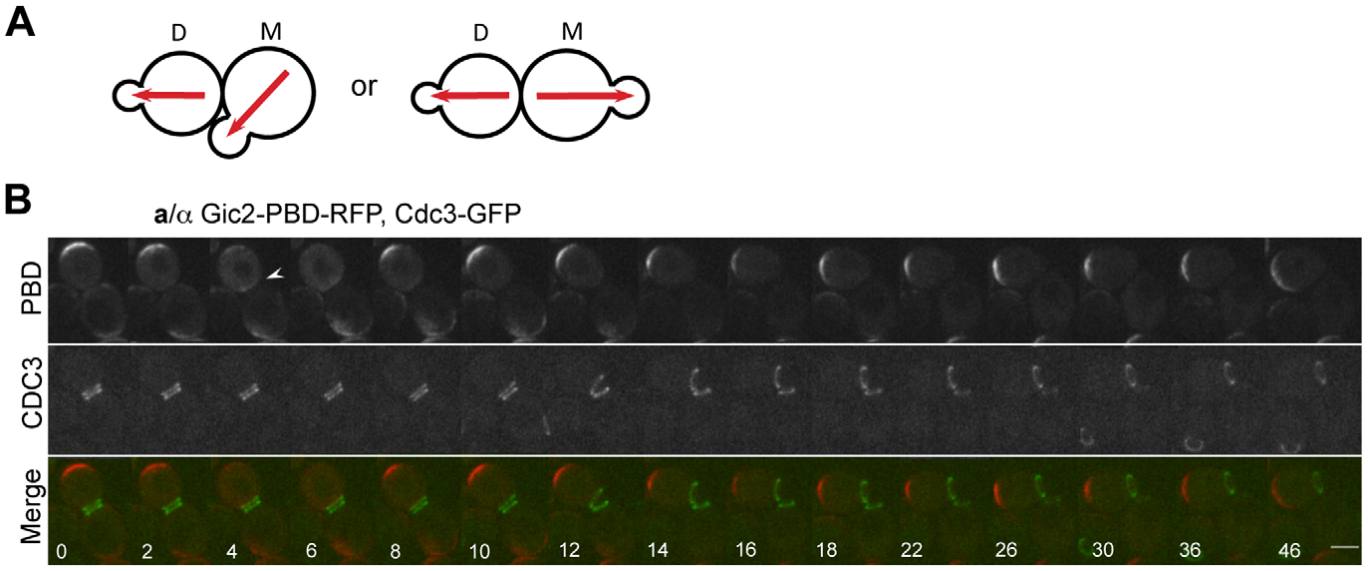
Time-lapse microscopy of Cdc42-GTP polarization in wild-type a/α diploids. **A.** A schematics diagram of the bipolar budding pattern. M and D stand for mother and daughter cells, respectively. Red arrows depict the axis of cell polarity. **B.** Localization of Gic2-PBD-RFP and Cdc3-GFP in diploid wild-type cells (HPY2353). An arrowhead marks Gic2-PBD-RFP localized to the proximal pole in the daughter cell. Numbers indicate time (in min) from the first image. Size bars, 3 µm.

Cell polarization is essential for a variety of cellular processes and functions. Cdc42 is highly conserved from yeast to humans and plays a central role in polarity establishment [1], [2]. The budding yeast *Saccharomyces cerevisiae* provides a unique model to study the development of cell polarity owing to its pronounced cell polarization during growth and its experimental tractability. During vegetative growth, yeast cells choose a specific bud site depending on their cell type, which determines the axis of polarized cell growth. Haploid **a** and α cells bud in the axial pattern, in which both mother and daughter cells select a new bud site adjacent to their immediately preceding division site. In contrast, **a**/α cells (normal diploids) bud in the bipolar pattern, in which daughter cells usually bud at the pole distal to the previous division site (distal pole) and mother cells can choose a new bud site near the proximal pole (birth pole) or the distal pole (see **Fig. 1A**) [3], [4], [5], [6]. These different budding patterns occur in response to cell-type-specific markers. The Rsr1 GTPase module, which is composed of Rsr1*/*Bud1, its GTPase activating protein (GAP) Bud2, and its GDP-GTP exchange factor (GEF) Bud5 [3], [7], [8], [9], [10], [11], links the spatial cues to the polarity establishment machinery including Cdc42. Cdc42 thus becomes polarized at the predetermined cortical site to trigger bud growth (see review [2] and references therein).

How do **a**/α cells select a bud site either at the distal or proximal pole? Previous studies uncovered a large number of genes affecting the bipolar budding pattern [12], [13], [14], [15]. These studies also indicate a close link between the cell cycle progression and the bipolar budding pattern [14], [16]. The bipolar pattern is dependent on transmembrane proteins including Bud8, Bud9, Rax1 and Rax2 [12], [17], [18], [19], [20]. Bud8 localizes to the distal pole of a newly born cell whereas Bud9 localizes to the bud side of the mother-bud neck (which becomes the proximal pole of a daughter cell) just before cytokinesis [20]. These localization patterns of Bud8 and Bud9 are consistent with their roles as putative distal and proximal pole markers, respectively. Rax1 and Rax2 localize to the tip of growing buds and to the mother-bud necks, and their presence at the division site is persistent throughout multiple generations [17], [18], [19]. Despite these interesting localization patterns of the putative bipolar landmarks, the mechanism by which the bipolar pattern is established remains largely unknown. One of the key questions is why daughter cells of **a**/α diploids choose predominantly the distal pole for their first budding despite the presence of Bud8 and Bud9 marking each pole. The complexity of the budding patterns led us to take a minimalist approach to address the question by combining mathematical modeling and live-cell imaging.

Recent studies in budding yeast have uncovered mechanisms by which Cdc42 becomes polarized in the absence of spatial cues via a process called 'symmetry breaking'. Two positive feedback mechanisms of symmetry breaking have been suggested – one involving the actin cytoskeleton and the other relying on a Cdc42 signaling network including the scaffold protein Bem1 and the Cdc42 GEF Cdc24 [21], [22], [23], [24]. Endocytosis- and GDI (Guanosine nucleotide dissociation inhibitor)-mediated recycling of Cdc42 and a negative feedback loop confer robust initiation of cell polarization [25], [26], [27], [28]. Using a stochastic mathematical model, an intrinsic stochastic mechanism involving linear positive feedback alone was shown to be sufficient to account for the spontaneous establishment of a single polarization site [29]. A Turing-type mechanism involving short-range excitation and long-range inhibition has also been proposed to explain the self-organized emergence of polarity [30], [31]. These models capture several features of cell polarization and provide a mechanistic insight into spontaneous polarization in the absence of spatial cues. However, some aspects of these mechanisms and their physiological relevance are still unclear and controversial [31], [32], [33]. More importantly, it had been unclear whether and how the spatial cues are recognized and amplified through these feedback mechanisms.

Here, we used computational modeling and live-cell imaging to explain cell polarization in diploid daughter cells. Because wild-type yeast cells undergo polarization in response to the cell-type-specific spatial cues, we considered these cues to understand distinct budding patterns. We report that both spatial landmarks and GTP hydrolysis of Cdc42 by Rga1 control the robust Cdc42-GTP polarization in diploid daughter cells.

## Results and Discussion

### A Mathematical Model of Cdc42 Polarization in Diploid Daughter Cells

Diploid **a**/α cells exhibit a strong bias toward the distal pole during their first and second bud-site selection [4], [12], [20] (**Fig. 1A**). To examine this preferential distal-pole budding event in daughter cells of diploids more closely, we monitored localization of Cdc42-GTP every 2 min in wild-type diploid cells expressing Gic2-PBD-RFP (tdTomato fused to the p21-binding domain of Gic2) as a reporter for Cdc42-GTP [34] and GFP fused to Cdc3, a component of septins, as a marker for the timing and site of cytokinesis. As expected, Gic2-PBD-RFP localized to the periphery of a growing bud until the end of the M phase, to the mother-bud neck (which becomes the proximal pole of daughter cells) during cytokinesis, and then to the distal pole in the daughter cells in the next G1 phase (100%, n = 15 movies) (**Fig. 1B**; **Movie S1**). While Cdc42 becomes enriched at the mother-bud neck at the division site [34], [35], the Gic2-PBD-RFP signal at the proximal pole was relatively weak presumably due to rapid hydrolysis of Cdc42-GTP by its GAP(s), consistent with a previous finding in haploids [34]. Nonetheless, our imaging was able to capture the daughter cells at an intermediate stage that exhibited Cdc42-GTP localization at both proximal and distal poles (see a cell marked with an arrowhead in **Fig. 1B**). The dynamics of Cdc42-GTP polarization is thus consistent with the distal-pole budding of diploid daughter cells.

**Figure 2:**
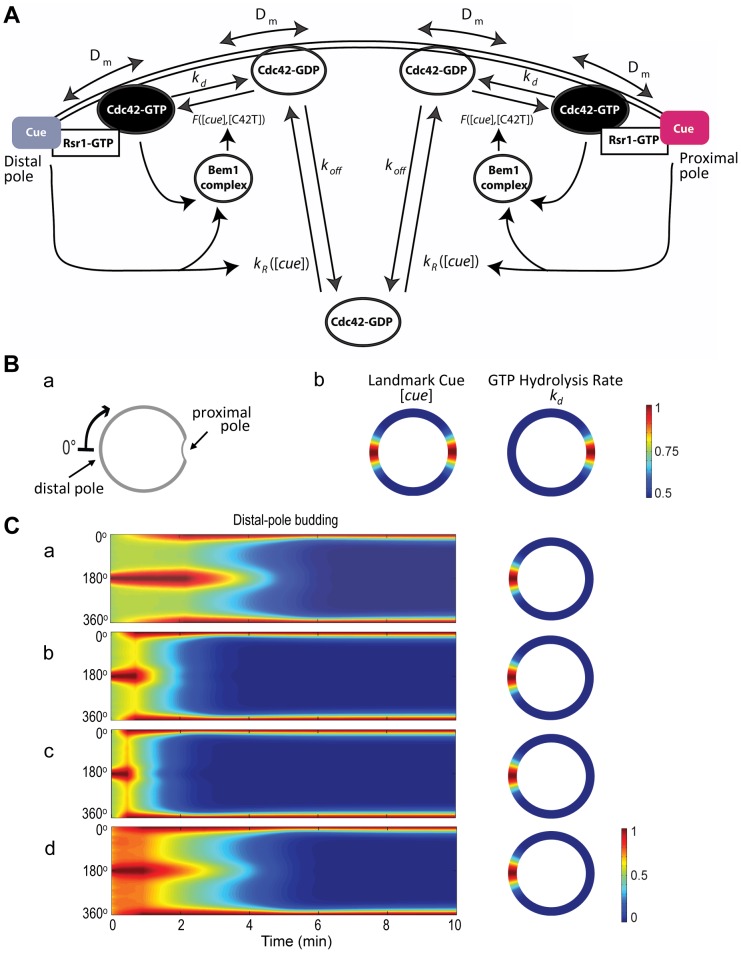
Mathematical modeling of Cdc42 polarization. **A.** A schematic diagram of the reaction-diffusion model with the following parameters: *D_m_* (the diffusion rate coefficient of Cdc42-GDP and Cdc42-GTP on the plasma membrane), *k_d_* (the inactivation rate coefficient of Cdc42 from the GTP- to the GDP-bound states), and *k_off_* (the rate at which the membrane-bound Cdc42-GDP is extracted into the cytoplasm). The function *F*([*cue*], [*C42T*]) represents the Bem1-mediated activation rate; and the function *k_R_*([*cue*]) is the landmark-signal-dependent recruitment rate of Cdc42 from the cytoplasm to the membrane. See details in Materials and Methods and the values of parameters in **Tables 1** and **2**. **Ba.** Coordinate of the periphery of an **a**/α daughter cell. The cell periphery is parameterized by the radial angle (0°–360°) in a clockwise direction starting from the distal pole. **Bb**. Spatial distributions of the landmark cues and the GTP hydrolysis rate of Cdc42. Both quantities are normalized by their maximal values for better visualization, with the color map scale shown on the right. **C.** Spatiotemporal dynamics of Cdc42-GTP in a diploid daughter cell. The cell periphery is presented as a 1D vertical axis, with the proximal pole in the middle (180°) and distal pole at the top/bottom (0°/360°). The horizontal axis represents the time window from 0 to 10 min. The localization of Cdc42-GTP at steady state is shown on the 2D cell periphery (right). The level of Cdc42-GTP is normalized by its maximal value at steady state, and the color map is displayed. See parameters in **Tables 1** and **2** for each simulation.

Why do daughter cells of diploids exhibit such dynamics of Cdc42-GTP despite the presence of spatial cues at both poles? Since our current knowledge of the bipolar landmark(s) does not provide a clear explanation for this time-evolved polarization of Cdc42-GTP in diploid daughter cells, we turn to mathematical modeling. We took into consideration several previous experimental observations and previous models for symmetry breaking. We assumed that the distal and proximal poles compete for Cdc42 or its effectors and regulators (**Fig. 2A**). Our model was built upon the positive feedback mechanism involving the Bem1 complex originally proposed by Goryachev and Pokhilko [30] and Lew and colleagues [24]. Importantly, our model included the Cdc42 GAPs to account for the weak Gic2-PBD-RFP localization at the division site and the spatial cues at both poles.

Specifically, several space-dependent rate parameters are included in our model as schematically shown in **Fig. 2A**. The establishment of Cdc42 polarization relied on the activation from its GDP- to GTP-bound state, which presumably depends on the pre-localized landmark signal and the Bem1-mediated feedback. This feedback was implemented in the activation rate of Cdc42 from the GDP- to the GTP-bound states (denoted by *F*), which depends on the levels of landmark cue (denoted by [*cue*]) and Cdc42-GTP, under the assumption that Bem1 is conserved (see Materials and Methods). The inactivation rate (*k_d_*) of Cdc42 from the GTP- to the GDP-bound states was space-dependent because it was assumed to vary with the level of the Cdc42 GAPs (which localize to the division site [34], [36], [37], [38]). The recruitment rate (*k_R_*) of Cdc42 from the cytoplasm to the membrane represents the association rate of cytoplasmic Cdc42-GDP with the membrane. The rate *k_R_* depends on the level of spatial cues because Rsr1 is likely to interact with Cdc42 to enhance its recruitment to the membrane in response to the landmark [39]. The landmark and the Rsr1 module were considered together as an upstream input to represent the spatial cue that triggers the initial localization of Cdc42. Thus *k_R_* was positively correlated with the level of the landmark signal in our simulations (see details in Materials and Methods). The parameters used in our simulations are listed in **Tables 1** and **2**.

**Table 1:** Ranges of parameters in the simulations.

Parameter	Value	Definition	Reference
*_R_*	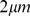	Radius of the cell	This study
*D_m_*	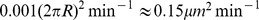	Lateral surface diffusion coefficient	[29], [30]
*k_off_*	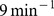	Rate coefficient from membrane to cytoplasm	[29]
*_k_*_Rec_	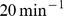	Rate parameter for recruitment from membrane to cytoplasm	[29]
*_KR_*	0.1	Parameter in recruitment rate	This study
*_kdL_*	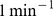	Inactivation rate coefficient of Cdc42	This study
*_kdH_*	_1∼2 min_^−1^		
*_kon_*	_0.1 min_^−1^	Activation rate coefficient of Cdc42	[30]
*C*_0_	0.1	Basal level of [*cue*]	This study
*C*_1_	0.15∼0.25	Level of [*cue*] at proximal pole	This study
*C*_2_	0.15∼0.25	Level of [*cue*] at distal pole	This study
*A*_0_	0.3	Initial level of [*C*42*D*]	This study
*A*_1_	0.005	Basal level of initial [*C*42*T*]	This study
*A_2_*	0.005∼0.01	Maximum level of initial [*C*42*T*]	This study
*K*	0.015	EC50 of the feedback	This study

**Table 2:** Specific parameters used for simulations.

	Fig. 2. Ca	Fig. 2. Cb	Fig. 2. Cc	Fig. 2. Cd	Fig. 7. Ab	Fig. 7. Ac, top	Fig. 7. Ac bottom	Fig. 7B, top	Fig. 7B, bottom
*kdH*	2 min^−1^	2 min^−1^	2 min^−1^	2 min^−1^	1 min^−1^	1 min^−1^	1 min^−1^	2 min^−1^	2 min^−1^
*C*_1_	0.25	0.2	0.15	0.25	0.2	0.2	0.15	0.25	0.1
*C*_2_	0.15	0.2	0.25	0.15	0.2	0.25	0.25	0.1	0.25
*A*_2_	0.01	0.01	0.01	0.007	0.01	0.007	0.005	0.01	0.01

Our model involved two reaction-diffusion equations to describe the spatial dynamics of Cdc42-GTP and Cdc42-GDP (Eq. [1]–[2] in Materials and Methods) on a cross section of the cell membrane with a diameter of 4 µm. In this model, the spatially distributed landmark [*cue*] was assumed to be a function of the membrane periphery, which was parameterized by the angle *x* along the circle (0° ≤ *x* ≤360°) from the distal pole (**Fig. 2B** **, a**). The function [*cue*] thus took maximal values locally at the proximal and distal poles (**Fig. 2B** **, b**) to represent the localized landmark at these poles. Our model also involved the following reactions: lateral membrane diffusion of Cdc42-GTP and Cdc42-GDP, activation of Cdc42 to the GTP-bound state and its inactivation, recruitment of Cdc42 from the cytoplasm to the membrane and its reverse reaction, and GDI-mediated extraction of Cdc42-GDP into the cytoplasm (see Materials and Methods).

Our simulations started with a homogeneous level of Cdc42-GDP at initial time *t = *0 and with Cdc42-GTP localized at the proximal pole of the cell, since Cdc42 is polarized to the division site (**Fig. 2C**). Fluctuations in the initial levels of these species due to naturally noisy background led to Cdc42-GTP clustering initially at both poles, which coexisted for a period of time. The Cdc42-GTP cluster at the proximal pole (180°) was gradually destabilized due to GTP hydrolysis by Cdc42 GAP(s) at the previous budding site, resulting in Cdc42 polarization at the distal pole. Indeed, the Cdc42-GTP cluster was consistently formed at the distal pole with any set of parameters within the ranges shown in **Table 1** (**Fig. 2C** **, a–d**), suggesting that the outcomes of competition are relatively insensitive to the concentration of spatial cues at each pole. Our modeling thus explains robust distal-pole budding of **a**/α daughter cells despite the competition between two poles for recruiting the Bem1 complexes and Cdc42-GTP.

### Deletion of *RGA1* Affects the Distal-pole Budding in Daughter Cells of a/α Diploids

**Figure 3:**
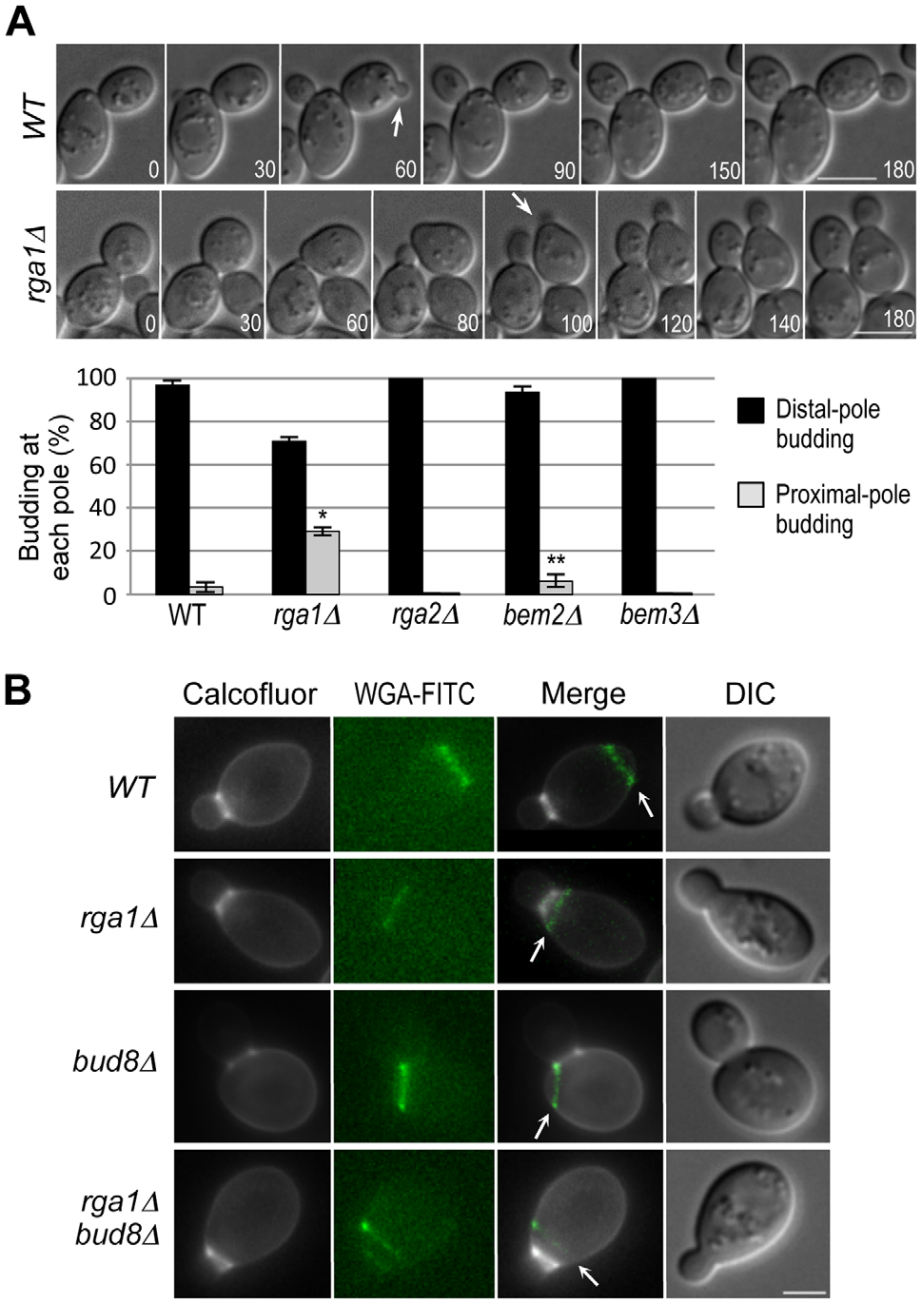
Positions of the first bud in a/α daughter cells of wild type and mutants deleted for Cdc42 GAPs. **A.** Time-lapse DIC images of diploid cells of wild type (YEF473) and *rga1Δ* (YEF1233). Arrows indicate budding events from daughter cells. Numbers indicate times (in min) from the first image. Size bars, 5 µm. (Histogram) The position of the first bud of daughter cells was scored in wild type (HPY1680), *rga1Δ* (HPY2205), *rga2Δ* (HPY2246), *bem2Δ* (HPY2384), and *bem3Δ* (HPY2426). The mean percentage ± SD of each budding pattern is shown from three or four independent countings of wild type (n = 106), *rga1Δ* (n = 144), *rga2Δ* (n = 56), *bem2Δ* (n = 108), and *bem3Δ* (n = 53). Statistical significance was determined by Student's t-test between proximal-pole buddings in wild type and *rga1Δ* or *bem2Δ* (marked with asterisks): *p<10^−5^ (*rga1Δ*) and **p = 0.02 (*bem2Δ*). **B.** The position of the first bud relative to the birth scar in diploid daughter cells. Cells were double stained with Calcoflour white and WGA-FITC as described in [44] from wild type (YEF473), *rga1Δ* (YEF1233), *bud8Δ* (YHH415), and *rga1Δ bud8Δ* (HPY2385). Arrows indicate birth scars. Size bar, 3 µm.

Because our modeling suggested that Cdc42 GTP hydrolysis rate at the division site contributes to robust distal-pole budding in **a**/α daughter cells, we wondered which Cdc42 GAP(s) play a role in this process. All predicted Cdc42 GAPs localize to the mother-bud neck at cytokinesis [34], [36], [37], [38]. We thus scored the position of the first bud of newly born daughter cells of diploid wild type and mutants deleted for a Cdc42 GAP such as Rga1, Rga2, Bem2, or Bem3. As expected, daughter cells rarely budded at the proximal pole in wild-type cells (3.4±2.3%, n = 106). In contrast, a significant number of daughter cells of an **a**/α *rga1Δ* homozygous diploid strain budded at the proximal pole (29.3±1.9%, n = 144; see daughter cells marked with arrows in **Fig. 3A**), which is statistically significant (p<10^−5^). Deletions of *RGA2* or *BEM3* did not result in proximal-pole budding in daughter cells (0%, n = 56 and 53, respectively). While a *bem2* deletion resulted in slightly increased proximal-pole budding (6.4±2.8%, n = 108), the difference between wild type and *bem2Δ* does not seem to be statistically significant (p = 0.22) (**Fig. 3A**). It is less clear whether Bem2, which is known as a GAP for Rho1, also functions as a GAP for Cdc42 *in vivo* [37], [40], [41], [42]. Taken together, these results suggest that among the Cdc42 GAPs, Rga1 is uniquely required for the preferential distal-pole budding of **a**/α daughter cells. We thus focused on Rga1 in subsequent studies.

Because Rga1 is uniquely required for preventing budding at the division site [34], we wondered whether the diploid *rga1Δ* daughter cells that failed to bud at the distal pole also budded at the division site. Unlike mother cells, which have bud scars (chitinous scar tissue located at the division site), daughter cells have a much less conspicuous birth scar (which has little or no chitin) at the division site [43]. To examine more closely the first bud position in daughter cells relative to birth scar, we stained cells with Calcofluor, which stains bud scars as well as the base of a bud, and FITC-labeled wheat germ agglutinin (WGA-FITC), which stains both bud scars and birth scars [44]. As expected, almost all wild-type daughter cells formed a bud opposite to the birth scar (which is marked with an arrow in **Fig. 3B**). In contrast, all of the *rga1Δ* daughter cells that failed to bud at the distal pole indeed budded within the birth scar (n = 65; note: this number includes some mother cells of *rga1Δ* because those mother cells that repeatedly budded within the birth scar could not be easily distinguished from daughter cells). As expected, almost all *bud8Δ* daughter cells budded at the proximal pole, but the position of a bud in *bud8Δ* was adjacent to, rather than within, the birth scar (97.4%, n = 39) (**Fig. 3B**). A small number of daughter cells of the diploid wild type (3.5%, n = 56) and *bem2Δ* mutant (6.3%, n = 63) also budded at the proximal pole, but these buds rarely appeared within the birth scar (data not shown). Interestingly, almost of all *rga1Δ bud8Δ* cells also budded within the birth scar (99.2%, n = 137; this counting is also likely to include some mother cells due to deletion of *RGA1*, see above). Taken together, these observations suggest that reduced distal-pole budding in the diploid *rga1Δ* daughter cells results from the increased Cdc42-GTP at the division site, consistent with a previous report [34].

### Polarization of Cdc42-GTP in Diploid Daughter Cells Lacking *RGA1*

**Figure 4:**
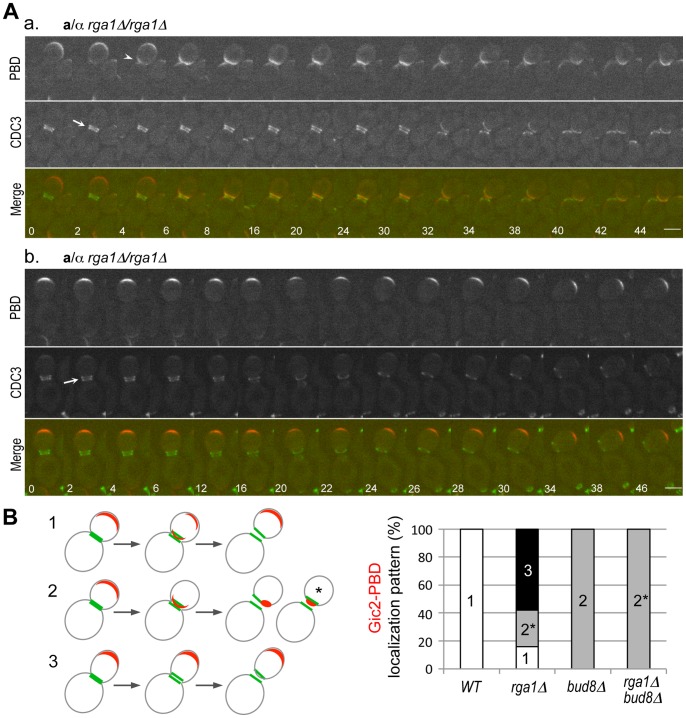
Localization of Gic2-PBD-RFP and Cdc3-GFP in *rga1Δ* cells. **A.** In *rga1Δ* cells (HPY2204), Gic2-PBD-RFP localized continuously to (a) the proximal pole or (b) the distal pole from cytokinesis to the next G1 phase. Arrows in (a) & (b) denote the Cdc3 ring splitting and an arrowhead in (a) denotes Gic2-PBD-RFP enriched at the division site (as well as the bud tip). Numbers indicate times (in min) from the first image. Size bars, 3 µm. **B.** Localization pattern of Gic2-PBD-RFP (red) prior to, during, and after cytokinesis (Cdc3-GFP in green) is summarized from time-lapse imagings of wild type (n = 15), *rga1Δ* (n = 19), *bud8Δ* (n = 7) and *rga1Δ bud8Δ* (n = 8). The proximal-pole localization pattern (marked with 2*) of *rga1Δ* or *rga1Δ bud8Δ* daughter cells is different from those seen in wild type and *bud8Δ* cells (see text for details).

Although some diploid *rga1Δ* daughters budded within the birth scar, the majority of them (∼70%) still showed strong preference for distal-pole budding. To gain insight into this cellular behaviour, we monitored the localization of Cdc42-GTP (using Gic2-PBD-RFP) in diploid *rga1Δ* cells every 2 min. Gic2-PBD-RFP localized to the periphery of a growing bud in an *rga1Δ* mutant as in wild type until cytokinesis. During cytokinesis and in the next G1 phase, however, three different patterns of Gic2-PBD-RFP localization were observed in *rga1Δ* daughter cells (n = 19 movies): 1) Gic2-PBD-RFP localized to the proximal pole and then to the distal pole, as seen in wild type (15.8%; not shown); 2) Gic2-PBD-RFP remained at the proximal pole (26.3%; **Fig. 4A** **, a**; **Movie S2**); and 3) Gic2-PBD-RFP continuously localized to the distal pole in a large percentage of daughter cells (57.9%; **Fig. 4A** **, b**; **Movie S3**). Both the first and third patterns of Cdc42-GTP localization were expected to lead to the distal-pole budding in **a**/α *rga1Δ* daughter cells (see summary in **Fig. 4B**). The localization patterns of Gic2-PBD-RFP are thus consistent with the observed budding patterns of the *rga1Δ* daughter cells (see **Fig. 3**).

While an increase of Cdc42-GTP at the proximal pole was expected given the lack of Cdc42 GAP activity at the division site in the *rga1Δ* mutant [34], it seemed counterintuitive that a significant percentage of *rga1Δ* daughter cells exhibited Cdc42-GTP polarization persistently at the distal pole. The one caveat is that our imaging was not fast enough to capture transient localization to the proximal pole in the third pattern (**Fig. 4B**). Nonetheless, these observations indicate that the dynamics of Cdc42-GTP in *rga1Δ* cells is different from that in wild type. Rga1 might thus have a unique role in Cdc42 polarization in diploid cells in addition to its role in clearing Cdc42-GTP at the division site (see below).

### Bud8 is Necessary for Polarization of Cdc42-GTP to the Distal Pole in Diploid *rga1Δ* Daughter Cells

**Figure 5:**
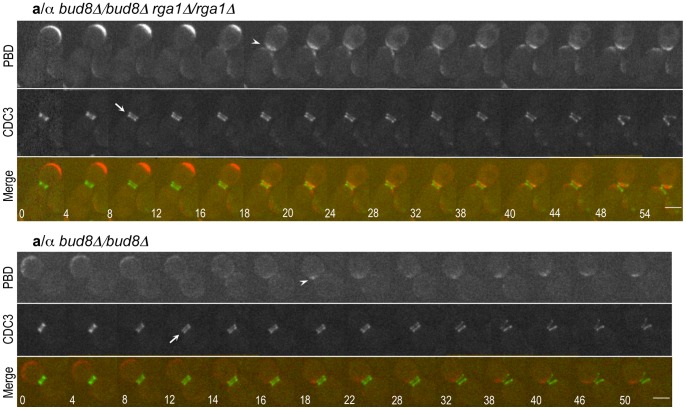
Localization of Gic2-PBD-RFP and Cdc3-GFP in the diploids homozygous for *bud8Δ rga1Δ* and *bud8Δ*. Imaging was performed as in Fig. 4 except in *bud8Δ* (HPY2370) and *rga1Δ bud8Δ* (HPY2371) cells. Arrows denote the Cdc3 ring splitting and arrowheads denote Gic2-PBD-RFP enriched at the proximal pole. Note: Gic2-PBD-RFP became enriched at a site adjacent to the Cdc3 ring in the *bud8Δ* daughter cell, whereas it appeared within the Cdc3 ring in *rga1Δ bud8Δ* daughter cell. Numbers indicate times (in min) from the first image. Size bars, 3 µm.

Since Bud8 functions as a distal pole marker important for normal bipolar budding pattern [20], we wondered whether the persistent distal-pole localization of Cdc42-GTP in the *rga1Δ* daughter cells is dependent on Bud8. Alternatively, Cdc42-GTP might be polarized to the distal pole independently of Bud8 as seen in the distal-pole budding of the *rsr1* mutant during haploid invasive growth [45]. To distinguish these possibilities, we examined the Gic2-PBD-RFP localization in cells lacking both *RGA1* and *BUD8* by time-lapse microscopy. While Gic2-PBD-RFP still localized to the periphery of growing buds prior to cytokinesis in the *rga1Δ bud8Δ* cells, it always localized to the proximal pole during cytokinesis and remained at the proximal pole in the *rga1Δ bud8Δ* cells (100%, n = 8 movies) (**Fig. 5** **, top panel; Movie S4**). This observation indicates that Bud8 functions as a spatial cue for the enrichment of Cdc42-GTP at the distal pole of the *rga1Δ* daughter cells as in wild-type cells. Interestingly, Gic2-PBD-RFP localized to a site within the old Cdc3 ring (*i.e*., within the birth scar) in the *rga1Δ bud8Δ* daughter cells. In contrast, Gic2-PBD-RFP localized to the division site at cytokinesis but subsequently to a site adjacent to the old Cdc3 ring in *bud8Δ* cells (100%, n = 7 movies) (**Fig. 5** **, bottom panel; Movie S5**). These Cdc42-GTP polarization patterns are thus consistent with the first bud positions in daughter cells of these mutants (see **Fig. 3B**).

Why is it that the persistent enrichment of Cdc42-GTP at the distal pole was observed only in *rga1Δ* daughter cells (see **Fig. 4B**)? How might Rga1 control Cdc42-GTP polarization? At the early phase of the cell cycle, most growth is targeted to the tip of the bud in budding yeast. This ‘apical’ growth is switched to ‘isotropic’ growth in the G2 phase, during which growth is distributed diffusely within the bud, and then cells are repolarized at the site of cytokinesis [46]. It has been suggested that apical growth and repolarization during cytokinesis are critical for establishing spatial cues at the distal and proximal poles, respectively, and thus subsequent positioning of the division plane in diploid cells [14]. The *rga1Δ* cells have elongated bud morphology [47], [48], [49], [50], suggesting a delay in the transition from apical growth to isotropic growth. We thus speculated that the prolonged apical growth of the *rga1Δ* mutant might result in more efficient delivery of the distal-pole marker such as Bud8 to the distal pole. To test the idea, we examined Bud8 localization.

**Figure 6:**
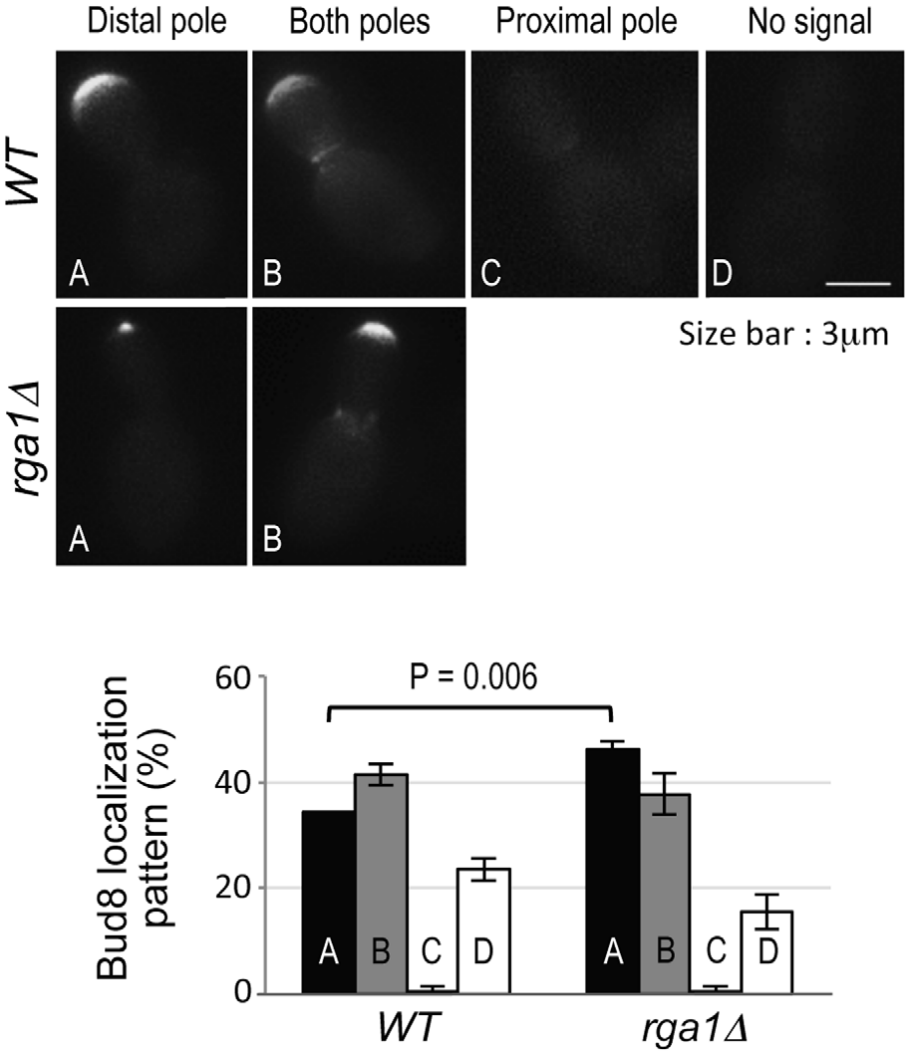
Localization of Bud8 in large-budded cells of the wild type (HPY1680) and *rga1Δ* (HPY2205) carrying YEpGFP-BUD8F. Representative images are shown for each pattern (A-D) and the percentage (mean ± SD) of each pattern is shown from three independent experiments (n = 160–230). Student's t-test was performed to compare the distal-pole localization in wild type and *rga1Δ* (P = 0.006).

Bud8 localized to the bud tip of growing buds and the distal pole of wild-type daughter cells after division, as previously reported [20]. A significant percentage of large-budded cells also exhibited Bud8-GFP localization at both bud tips and the bud side of the mother-bud neck, although the latter was often weaker [19], [20], [51]. Interestingly, more large-budded *rga1Δ* cells exhibited Bud8-GFP localization to the bud tip (46.2±1.5%, n = 165) compared to wild type (34.5±0.1%, n = 174) (**Fig. 6**), and this difference appeared to be statistically significant (p = 0.006). Bud8-GFP often appeared to be confined to the extreme bud tip in these *rga1Δ* cells (**Fig. 6**). A minor difference of Bud8 localization was also observed in unbudded cells of *rga1Δ* compared to wild type (data not shown). These observations are thus consistent with the idea that Bud8 is more efficiently targeted to the bud tip (which becomes the distal pole of daughter cells) in *rga1Δ* cells, perhaps due to longer apical growth. However, it is unclear whether this different pattern of Bud8 localization solely accounts for persistent Cdc42-GTP polarization to the distal pole of *rga1Δ* cells. Indeed, we observed robust Cdc42-GTP polarization at the bud tip in large-budded cells of the *bud8Δ rga1Δ* mutant until cytokinesis (and even in *bud8Δ* cells, although Gic2-PBD-RFP appeared more broadly at the periphery of the buds in these cells) (see **Fig. 5**), suggesting that this Cdc42-GTP polarization prior to cytokinesis is independent on Bud8. Rga1 might also affect the targeting of Bud9 to the proximal pole or a component of the polarisome such as Spa2 or Ste20 at the bud tip [2], [14], which might affect Cdc42-GTP polarization prior to cytokinesis via a feedback mechanism (see below). Further investigation is necessary to understand the underlying mechanism involved in polarized growth and selection of a growth site in diploids.

### Modeling Predicts Different Dynamics of Cdc42 Polarization Depending on the Levels of Spatial Cues as well as the GTP Hydrolysis of Cdc42

**Figure 7:**
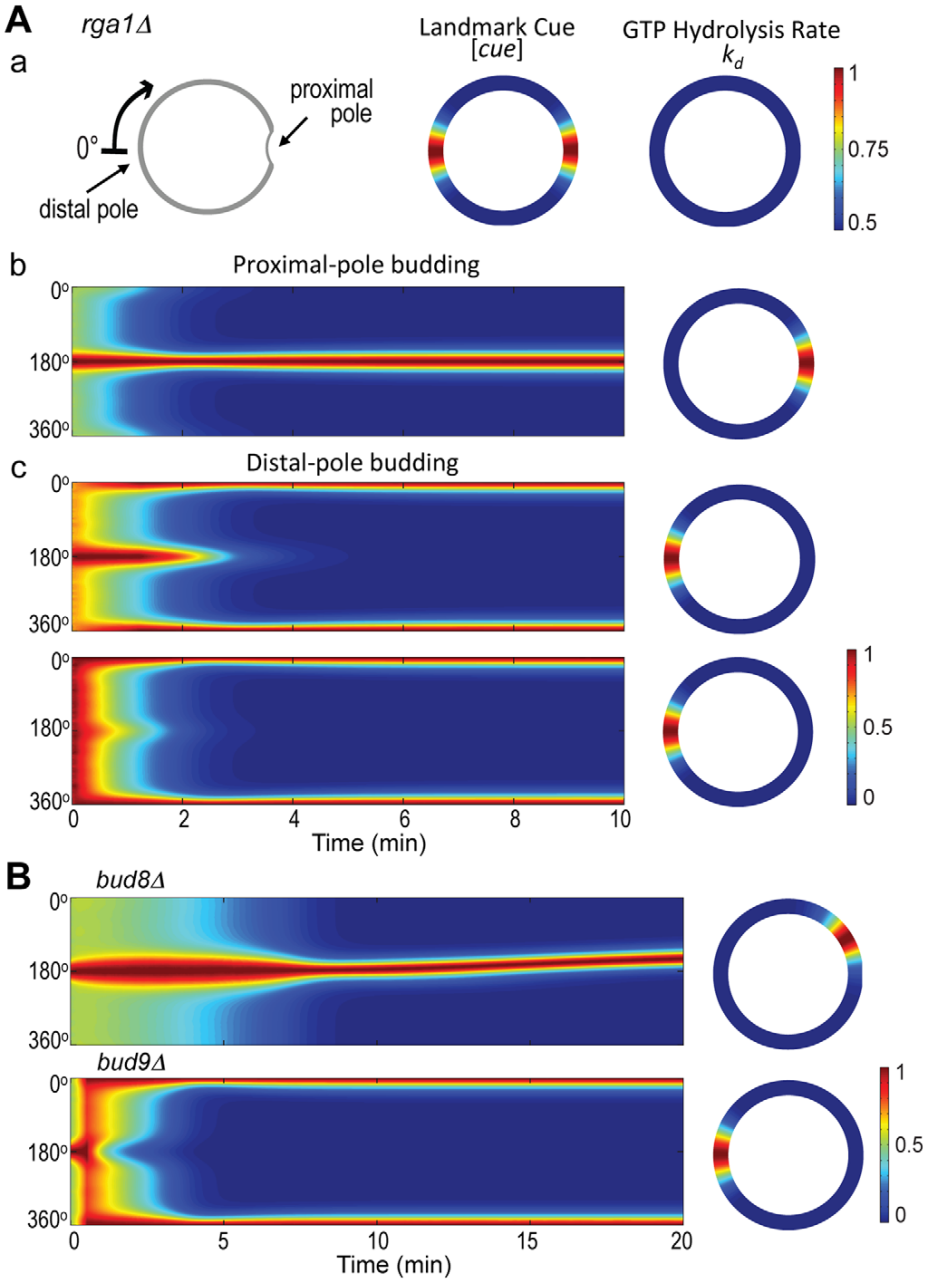
Mathematical modeling of Cdc42 polarization in diploid daughter cells deleted for *RGA1*, *BUD8* or *BUD9*. **Aa.** Coordinates are the same as in Fig. 2 (wild type) but the GTP hydrolysis rate of Cdc42 in the *rga1Δ* mutant is assumed to be about the same along the perimeter. See parameters in Table 2. **Ab–Ac.** Spatiotemporal dynamics of Cdc42-GTP leading to budding at (b) the proximal pole or (c) the distal pole in *rga1Δ* daughter cells. The horizontal axis represents the time window from 0 to 10 min. The 2D steady-state distribution of Cdc42-GTP is displayed on the right to each simulation. **B.** Spatiotemporal dynamics of Cdc42-GTP in diploid *bud8Δ* (top) and *bud9Δ* (bottom) mutants. The horizontal axis represents the time window from 0 to 20 min. The 2D steady-state distribution of Cdc42-GTP is displayed on the right to each simulation. Note: Cdc42-GTP became polarized at a site adjacent to the center of the proximal pole in *bud8Δ* (see 20 min time point), unlike in *rga1Δ* (see Fig. 7A, b).

We then asked whether our mathematical modeling could account for these different types of Cdc42-GTP dynamics in the absence of a Cdc42 GAP or the spatial cues. In the absence of Rga1, the GTP hydrolysis rate *k_d_* would be spatially uniform; i.e., Cdc42 activity was no longer inhibited at the proximal pole (**Fig. 7A** **, a**). We thus expected that Cdc42 was able to form a cluster at both proximal and distal poles marked by the landmarks and that its subsequent dynamics would be determined by the initial level of Cdc42-GTP at the division site and landmark cues that are subject to random perturbation. Our simulation showed that *rga1Δ* daughter cells could indeed bud at both poles: First, the high initial localization of Cdc42-GTP inhibited the formation of cluster at the distal pole, so that only one cluster was formed at the proximal-pole budding in the entire process (**Fig. 7A** **, b**). Second, if the initial localization of Cdc42-GTP to the division site was reduced and the level of the distal-pole landmark was increased, the Cdc42-GTP cluster eventually formed at the distal pole (**Fig. 7A** **, c**). Interestingly, the time window when Cdc42-GTP localized to both proximal and distal poles changes depending on the initial level of Cdc42-GTP at the division site and the strength of landmark cues. Within some parameter range that strength of landmark cue at the proximal pole is slightly less than that at the distal pole (see **Table 2**), Cdc42-GTP localization coexisted at both poles for a substantial time window (**Fig. 7A** **c, top**). However, when the ratio of strength of landmark cue at the distal pole to that at the proximal pole increases beyond that range, Cdc42-GTP localization to the proximal pole could be barely monitored (**Fig. 7A** **c, bottom**), and this scenario would account for the persistent distal-pole localization of Cdc42-GTP observed in over 50% of the *rga1Δ* daughter cells (see above). Our simulations thus predict that the relatively higher landmark at the distal pole or lower landmark at the proximal pole in the absence of Rga1 might result in persistent distal pole budding. These different patterns may thus arise from natural variations in the efficiency of delivery of these cues to the poles; in other words, the level of landmark cue or initial level of Cdc42-GTP in our model may be subject to substantial perturbation so that the parameters could fall in various ranges. While the exact mechanism remains unknown, a negative feedback loop involving Rga1 might be involved to buffer the level of Cdc42-GTP and thus to stop the polarity cluster from growing too large, as recently suggested by Howell et al. [28].

Next, we asked whether our modeling could recapitulate the behavior of *bud8Δ* and *bud9Δ* mutants, which bud exclusively at the proximal and distal poles, respectively [12]. We used similar parameters except that the landmark cue [*cue*](*x*) is high only at either the proximal or distal pole in *bud8Δ* or *bud9Δ*, respectively. Our simulations indeed indicated that Cdc42-GTP polarized to the proximal pole in a *bud8Δ* mutant (**Fig. 7B** **, top**) and to the distal pole in a *bud9Δ* mutant (**Fig. 7B** **, bottom**). It is noteworthy that Cdc42-GTP polarization developed eventually at a site adjacent to the division site in *bud8Δ*, unlike that in *rga1Δ*, consistent with the bud position in the daughter cells of these mutants (see **Figs. 3B** & **5**). Taken together, our computational modeling indicated different dynamics of Cdc42-GTP polarization when the levels of landmark and Cdc42-GTP were perturbed by noise in the model.

In summary, our mathematical modeling with limited parameters predicted the dynamics of Cdc42-GTP polarization, which accounts for robust distal-pole budding in diploid daughter cells. Live-cell imaging indicates that the distal-pole budding was dependent on Bud8 and GTP hydrolysis of Cdc42 by Rga1. While further investigation is necessary to fully understand the underlying mechanism, this study suggests that a Cdc42 GAP, not only the distal and proximal pole markers, affects the dynamics of Cdc42 polarization, contributing to selection of a growth site in diploid daughter cells.

## Materials and Methods

### A Mathematical Model of Cdc42 Polarization in Response to the Landmark Cues in Diploid Daughter Cells

The dynamics of the Cdc42-GTP and Cdc42-GDP on the cell membrane, with their particle densities denoted by [*C*42*T*] and [*C*42*D*], respectively, can be described by reaction-diffusion equations:
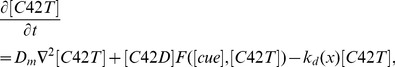
(1)

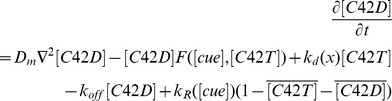
(2)


The terms 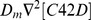 and 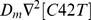 represent the surface lateral diffusion of Cdc42-GDP and Cdc42-GTP on the cell membrane, with 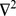 being the surface diffusion Laplacian operator and *D_m_* the diffusion rate. The level of the landmark cues ([*cue*]) is a function of the angle *x* which parameterizes the membrane periphery (0° ≤ *x* ≤360°) from the distal pole:
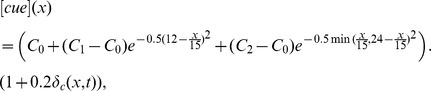
Where *δ_c_*(*x*,*t*) is a random variable from standard normal distribution to model the fluctuations from natural background. We remark here that in the absence of random fluctuation, [*cue*](*x*) is a function with basal level *C*_0_ and has two peaks, with maximal levels *C*_1_ and *C*_2_, at the proximal and distal poles, respectively. Other choices of the functional form with the same property will lead to similar results.

The Bem1-mediated feedback, implemented by the activation rate *F*, takes the form:
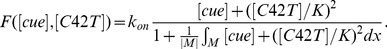
(3)


In equation [3], |*M*| denotes the total area of membrane surface, and the integral is taken over the cell membrane *M*, while the denominator represents the conservation of the total amount of Bem1 complex. We assume that the dynamic of Bem1 complex is much faster than that of Cdc42. We obtain the particle density of Bem1 complex at every time *t* by considering the quasi steady state solution of particle density of Bem1 complex, which is equal to the term
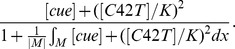



The detailed derivation can be found in the next section. In equations [1] and [2], the parameter *k_d_* represents the inactivation rate of Cdc42 from the GTP- to the GDP-bound states, which is space-dependent because it varies with the level of the Cdc42 GAPs. We define it to be in the following form, with higher level at the proximal pole (at 180°):
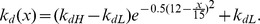



The parameters *k_dH_* and *k_dL_* are the maximal and minimal inactivation rates. **Fig. 2B** **b** shows the spatial distribution of [*cue*] and the GTP hydrolysis rate in wild type **a**/α daughter cells, in which *k_dH_* is assumed to be much larger than *k_dL_*. In **Fig. 7A**, *k_dH_* is taken to be equal to *k_dL_*, and therefore *k_d_* appears constant.

In equation [2], 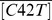 and 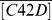 respectively represent the average amount of [*C*42*T*] and [*C*42*D*] over the membrane, that is, the integral of [*C*42*T*] and [*C*42*D*] over the cell membrane divided by the cell surface area. Thus, the recruitment of Cdc42 from the cytoplasm to the membrane is modeled by 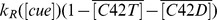, where *k_R_*([*cue*]) is the landmark-signal-dependent coefficient and 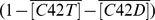 stands for the fraction of the cytoplasmic Cdc42. We remark here that to ensure 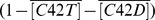 being between 0 and 1 to represent a fraction, the initial value for 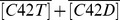 needs to be less than 1, which is true with the initial conditions and the associated parameters used in our simulations. Here we also assume that Cdc42 is uniformly distributed throughout the cytoplasm because cytoplasmic Cdc42 diffuses fast enough to reach a homogeneous state. In our simulations, we define the spatial-cue-dependent parameter *k_R_*([*cue*]) to be a function positively correlated with the function [*cue*], so that it has a similar spatial profile as the landmark cue. We choose to use the Michaelis-Menten form with power 1:
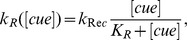
however, other function forms of *k_R_*, if properly scaled, can also produce the same results.

The parameter *k_off_* stands for the rate at which the membrane-bound Cdc42-GDP is extracted into the cytoplasm. This extraction of Cdc42-GDP away from the membrane is GDI-mediated, thus counteracting the recruitment of Cdc42.

For the initial values of our simulations, we assume that initially Cdc42-GDP is a constant and Cdc42-GTP is localized at the proximal pole of the cell, both of them with 20% perturbation from their basal levels. The initial values of [*C*42*D*] and [*C*42*T*] are defined as follows:
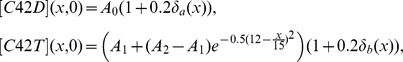
where *δ_a_*(x) and *δ_b_*(x) are the random variables from a uniform distribution between 0 and 1, and *A*_0_ is the basal level for Cdc42-GDP, while *A*_1_ and *A*_2_ are the basal maximal and minimal levels for Cdc42-GTP. All the above parameters are listed in **Tables 1** and **2**.

### Derivation of Equation [3]

Let *C*(*x,t*) denote the particle density of Bem1 complex on the cell membrane, which is governed by
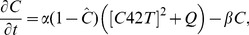
(4)where *α* and *β* are constant parameters; 
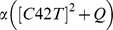
 is the recruitment rate of Cdc24 from cytoplasm to membrane, depended on the particle density of Cdc42; *Q* is the spatial function representing the level of stimulation by the landmark cue and *αQ* represents the basal recruitment rate controlled by the landmark cue; 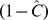 is the fraction of the cytoplasmic Cdc24; *βC* is the disassociation rate of *C* from the membrane to the cytoplasm; 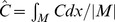 represents the average value of *C* over the membrane.

The dynamic of Bem1 complex is much faster than that of Cdc42. In the system of Cdc42, we obtain the particle density of Bem1 complex at every time *t* by solving the quasi-steady-state solution of the equation [4]. By assuming right hand side of [4] to be zero, the steady state equation of [4] can be written as follows:
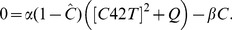
(5)


By taking the average value of right hand side of [5] over the membrane (taking integration over the membrane and then dividing by the area of the membrane), we have
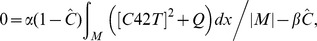
which leads to



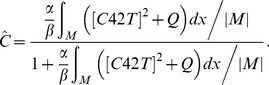
(6)By substituting [6] into [5], we can obtain *C* in term of [*C42T*]:
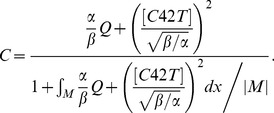
(7)


By defining [*cue*] = *αQ*/*β* and 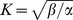, *C* can be rewritten into the form.
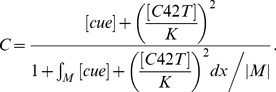
(8)


If we assume that the activation rate of Cdc42 is proportional to the quasi-steady-state solution of particle density of Bem1 complex and define [*cue*] = *αQ*/*β*, the form of the activation rate of Cdc42 will be



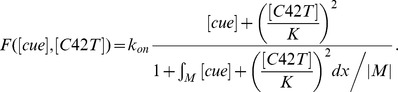
.

### Parameter Estimation

For simplicity, we considered a diploid daughter cell as a 4 µm-diameter circle, since daughter cells are generally smaller than mother cells, which are typically 5×6 µm ellipsoids. For a yeast cell of radius 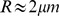, the membrane diffusion coefficient of Cdc42 is estimated to be.


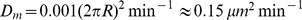
 [29]. According to [29], we estimate an off-rate from membrane to cytoplasm to be 
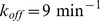
. Yeast cell polarization is mainly achieved by Bem1-mediated positive feedback and landmark cue is just an initial tracker for polarization so the level of landmark cue should be small comparing with feedback strength. Here we took the basal level of landmark cue to be *C*_0_ = 0.1 and *C*_1_, *C*_2_ = 0.15–0.25 which were small comparing with feedback strength we observed in simulations. The recruitment rate was estimated to be 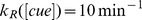 [29]. According to the definition of 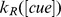, we took 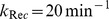 and 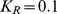. For the activation rate coefficient of Cdc42, we took 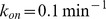 [30]. We assumed that the number of Cdc42-GTP on membrane is much smaller than total number of Cdc42, and thus we took inactivation rate coefficient 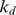 of Cdc42 to be between 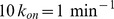 and 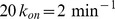.

### Numerical Method for Simulations

The simulations used a second-order central difference approximation for the diffusion terms, and the temporal discretization was carried out using a fourth order Adams-Moulton predictor-corrector method. FORTRAN 77 was used for the simulation shown in Figures 2 and 7 and plots were generated using MATLAB 7.

### Strains, Plasmids and Genetic Methods

Standard methods of yeast genetics, DNA manipulation, and growth conditions were used [52], [53] unless indicated otherwise. Plasmids YIp211-GIC2-PBD-1.5tdTomato and YIp128-CDC3-GFP (kindly provided by E. Bi, University of Pennsylvania) were used to construct strains expressing Gic2-PBD-RFP and Cdc3-GFP, respectively, as previously described [34]. Plasmids pRS314-HO and YCp50-HO (from the Park lab collection), which carry the *HO* gene, were used to generate **a**/α diploids. See **Table 3** for a list of strains used in this study.

**Table 3:** Yeast strains used in this study.

Strain		Relevant Genotype^a^	Source/Comments
YEF473*	**a**/α	*his3-* ***Δ*** *200/his3-* ***Δ*** *200 leu2-* ***Δ*** *1/leu2-* ***Δ*** *1 lys2-801/lys2-801 trp1-* ***Δ*** *63/trp1-* ***Δ*** *63 ura3-52/ura3-52*	[54]
YEF1233*	**a**/α	*rga1* ***Δ*** *::HIS3/rga1* ***Δ*** *::HIS3*	[34]
YHH415*	**a**/α	*bud8-* ***Δ*** *1::TRP1/bud8-* ***Δ*** *1::TRP1*	[20]
HPY2353*	**a**/α	*CDC3-GFP::LEU2/CDC3-GFP::LEU2 GIC2-PBD-RFP::URA3/GIC2-PBD-RFP::URA3*	This study
HPY2204*	**a**/α	*rga1* ***Δ*** *::HIS3/rga1* ***Δ*** *::HIS3 CDC3-GFP::LEU2/CDC3-GFP::LEU2 GIC2-PBD-RFP::URA3/GIC2-PBD-RFP::URA3*	This study
HPY2370*	**a**/α	*bud8-* ***Δ*** *1::TRP1/bud8-* ***Δ*** *1::TRP1 CDC3-GFP::LEU2/CDC3-GFP::LEU2 GIC2-PBD-RFP::URA3/GIC2-PBD-RFP::URA3*	This study
HPY2371*	**a**/α	*rga1* ***Δ*** *::HIS3/rga1* ***Δ*** *::HIS3 bud8-* ***Δ*** *1::TRP1/bud8-* ***Δ*** *1::TRP1 CDC3-GFP::LEU2/CDC3-GFP::LEU2* *GIC2-PBD-RFP::URA3/GIC2-PBD-RFP::URA3*	This study
HPY2385*	**a**/α	*rga1* ***Δ*** *::HIS3/rga1* ***Δ*** *::HIS3 bud8-* ***Δ*** *1::TRP1/bud8-* ***Δ*** *1::TRP1*	This study
HPY1680@	**a**/α	*his3-* ***Δ*** *1/his3-* ***Δ*** *1 leu2* ***Δ*** *0/leu2* ***Δ*** *0 met15* ***Δ*** *0/met15* ***Δ*** *0 ura3* ***Δ*** *0/ura3* ***Δ*** *0*	Diploid of BY4741 (Open Biosystems)
HPY2205@	**a**/α	*rga1* ***Δ*** *::kanMX4*/*rga1* ***Δ*** *::kanMX4*	This study
HPY2246@	**a**/α	*rga2* ***Δ*** *::kanMX4*/*rga2* ***Δ*** *::kanMX4*	This study
HPY2384@	**a/**α	*bem2* ***Δ*** *::kanMX4*/*bem2* ***Δ*** *::kanMX4*	This study
HPY2426@	**a/**α	*bem3* ***Δ*** *::KanMX4/bem3* ***Δ*** *::KanMX4*	This study

a Strains marked with *are isogenic to YEF473 and strains marked with ^@^are isogenic to HPY1680 except as indicated.

### Determination of the Budding Pattern and Localization of Bud8

To determine budding patterns, cells were spotted on a YPD plate after a brief sonication and then the position of each bud was monitored under the dissecting microscope at 25^o^C. For time-lapse imaging by DIC microscopy, cells were grown similarly, spotted on a slab of YPD medium containing 1% agarose, and then imaged using a Nikon E800 microscope (Nikon, Tokyo, Japan) fitted with a 100X oil-immersion objective (NA = 1.30) with a Hamamatsu ORCA-2 CCD camera (Hamamatsu Photonics, Bridgewater, NJ) and Slidebook software (Intelligent Imaging Innovations, Denver, CO) at 25^o^C. Localization of Bud8 was examined as previously described [19] using YEpGFP-BUD8F [16].

### 3D Time-lapse Microscopy

To visualize GFP- and RFP-fusion proteins, a slab of SC-Ura was prepared as above using exponentially growing cells in SC-Ura media. Images were captured at 23–24^o^C every 2 min using a spinning disk confocal microscope (UltraView ERS, Perkin Elmer Life and Analytical Sciences, Waltham, MA) equipped with a 100×/1.4 NA objective lens (Nikon, Melville, NY), a 488-nm solid state laser and 568-nm argon ion laser, and a cooled charge-coupled device camera (ORCA-AG, Hamamatsu, Bridgewater, NJ). Maximum intensity projections of Z-sections (spaced at 0.4–0.5 µm) are generated using UltraView ERS software. All time-point images are shown in Movies S1, S2, S3, S4, S5, and the selected time-point images are shown in Figs. 1, 4, and 5.

## Supporting Information

Movie S1 Localization of Gic2-PBD-RFP (left) and Cdc3-GFP (right) in **a**/α wild-type cells.
(AVI)

Movie S2 Localization of Gic2-PBD-RFP (left) and Cdc3-GFP (right) in **a**/α *rga1Δ* cells.
(AVI)

Movie S3 Localization of Gic2-PBD-RFP (left) and Cdc3-GFP (right) in **a**/α *rga1 Δ* cells.
(AVI)

Movie S4 Localization of Gic2-PBD-RFP (left) and Cdc3-GFP (right) in **a**/α *rga1 Δ* *bud8D* cells.
(AVI)

Movie S5 Localization of Gic2-PBD-RFP (left) and Cdc3-GFP (right) in **a**/α *bud8 Δ* cells.
(AVI)
